# Identification of sex-biased MiRNA markers informative of heat-past events

**DOI:** 10.1186/s12864-025-11551-8

**Published:** 2025-05-08

**Authors:** Tosca A. van Gelderen, Jerome Montfort, José Antonio Álvarez-Dios, Francesc Piferrer, Julien Bobe, Laia Ribas

**Affiliations:** 1 Institut de Ciències del Mar, Consejo Superior de Investigaciones Científicas (ICM-CSIC), Barcelona, 08003 Spain; 2 PhD Program in Genetics, Autonomous University of Barcelona, Bellaterra, 08193 Spain; 3 INRAE, Laboratoire de Physiologie et Génomique des Poissons, Rennes, France; 4 Departamento de Matemática Aplicada, Facultad de Matemáticas, Universidad de Santiago de Compostela, Santiago de Compostela, 15781 Spain

**Keywords:** European sea bass, High temperature, Evolution, Climate change, Fish

## Abstract

Elevated temperatures during early developmental stages play a pivotal role in the fate of the adult sexual phenotype of fish populations, usually leading to male-skewed sex ratios. This is the case with European sea bass (*Dicentrarchus labrax*), one of the most important species in the European aquaculture industry. To unveil informative markers of the past thermal events, we investigated changes in the miRNome within the gonads of this species. Consequently, we exposed European sea bass to elevated temperatures (21ºC) during early development (from 7 to 68 days post fertilization). After one-year post-heat treatment growing at natural temperature, a miRNA-sequencing analysis was conducted in the ovaries and testes of juvenile fish. The examination of miRNA expression levels identified three and twelve miRNAs in ovaries and testes, respectively, reflecting past thermal events. To assess the evolutionary conservation of these identified miRNAs in gonads, we cross-referenced our data with miRNome public information from ovaries and testes in nine additional fish species from the FishmiRNA database. This analysis uncovered 33 potential sex-biased markers present in at least five studied species along the evolutionary timeline. For instance, miR-155, miR-429, and miR-140 were consistently female-skewed, while miR-143, miR-499, and miR-135b-3p were consistently male-skewed. In addition, among these markers, three conserved sex-skewed miRNAs proved to be informative regarding past thermal events in the ovaries (e.g., miR-192-5p, miR-146a-5p and miR-143-3p) and four in the testes (miR-129-5p, miR-724-5p, miR-143-3p, and miR-223-3p). Notably, miR-223-3p was conserved female-skewed, but showed upregulation in males exposed to high temperature, and miR-143-3p was inhibited in both heated females and males. These miRNAs could serve as markers of heat-induced masculinization. This research broadens the inventory of sex-specific miRNAs across evolution in fish, and elucidates thermosensitive miRNAs in the gonads.

**Supplementary Information:**

The online version contains supplementary material available at 10.1186/s12864-025-11551-8.

## Introduction

The European sea bass (*Dicentrarchus labrax*) is important for aquaculture. Since 2016, aquaculture has accounted for 96% of the total European sea bass production in comparison with that obtained from fisheries [1, 2]. To ensure successful growth and development in cultured fish, it is essential to maintain optimal rearing. For economic production efficiency, European sea bass females are the desired sexual phenotype as they are favored due to their larger size, which can be up to 30% larger than their male counterparts [3]. However, farmed European sea bass is known to produce populations consisting of 70–100% males, due to increased temperatures during early stages of development in the hatcheries [4, 5]. Various studies have attempted to identify the ideal rearing temperature for European sea bass larvae but it is known that temperatures exceeding 16 °C during the thermal sensitive window (∼ 0 to 150 days post fertilization, dpf) lead to male-skewed populations [6, 7].

Unlike species with genotypic sex determination, some teleost fish, including the European sea bass, exhibit polygenic sex determination (PSD) [8]. In PSD, environmental factors like temperature [9], stress [10], hypoxia [11], density [12], chemical compounds [13] and even the activation of the immune system [14] play a substantial role in shaping the final sexual phenotype of individuals. These external influences impact gene expression through various epigenetic mechanisms (i.e., histone modification, DNA methylation and, post-transcriptional regulators, like miRNAs) [15]. The first description of epigenetics in sex regulation due to temperature in fish was given in European sea bass more than a decade ago. DNA methylation of the gonadal aromatase cytochrome P450, family 19, subfamily A (*cyp19a1a*) promoter —a key gene for ovarian development in non-mammalian vertebrates— was altered in adults previously reared at a high temperature during early development, favoring population masculinization [16]. Since then, research has been deployed to better understand the connection between the epigenome and sexual phenotypes. This is the case, for example, in pufferfish (*Takifugu rubripes*), or in half-smooth tongue sole (*Cynoglossus semilaevis*) [17, 18].

The function of miRNAs —small non-coding RNAs that play a vital role in post-transcriptional gene regulation— is to act as an epigenetic mechanism and serve as a bridge between the environment and the phenotype. In the last years, the importance of miRNAs in fish has flourished and thus research related to miRNA in cultured fish has grown exponentially. Many studies regarding temperature alterations and miRNA expression have been described in various species and tissues in fish. For example, after short-term and acute high or low temperature exposures (18 days), 29 miRNAs were differentially expressed (DE) in the common carp (*Cyprinus carpio*) liver [19]. In the Antarctic notothenioid (*Trematomus bernacchii*), adapted to extreme climates of temperatures around − 1 °C, an acute heat stress was able to alter twelve miRNAs in the gills, mainly pertaining to cellular stress response [20]. Long-term effects on the miRNome were shown in zebrafish (*Danio rerio*) [21] and in Atlantic cod (*Gadus morhua*) [22]. Further, adult zebrafish exposed to high temperature for 21 days changed the miRNome with a consecutive recovery in which miRNA expressions returned to their original state [23]. In particular, in European sea bass exposed at high temperature during different stages of early development, miRNA expression was assessed, showing an alteration of heat-sensitive miRNAs in which the immune and the reproductive systems were involved [24].

One of the particularities that makes miRNAs good markers for conserved developmental processes is their high level of conservation across evolution —sequence might be conserved but function could change across evolution— [25]. miRNAs emerged during early evolution, i.e., in metazoans, and some miRNAs such as miR-100, miR-125 and let-7 have been described as ancient miRNAs in bilaterians [26]. Thus, ever since their initial discovery, miRNAs have been used as tools in extensive applications in human health diagnostics, primarily for tumor detection [27]. In the context of aquaculture, and since the first miRNA discovery in zebrafish [28], numerous studies have been undertaken to explore and characterize the miRNA repertoire in other teleost species. In fish, miRNA expression patterns were proposed as indicators to improve productivity by, for example, optimizing selection for breeding programs. This is the case of rainbow trout (*Onchorhynchus mykiss*), in which circulating miRNAs in the blood were recently identified as a non-invasive approach to detail the metabolic and reproductive states [29]. In Nile tilapia (*Oreochromis niloticus*) miR-1, miR-206, and miR-133a abundance was interpreted as markers to link genetic polymorphism data with miRNA targets [30]. By using public databases, a set of miRNAs (i.e., miR-9-3p, miR-135c, miR-9-5p, miR-30b, miR-122 and miR-92a-3p) was suggested as a collection of potential markers for cold tolerance in fish [31].

Notwithstanding the existence of many studies regarding the miRNA roles in the reproductive system, little is still known about the presence of many miRNAs in fish, particularly in the context of sex development. The elucidation of conserved miRNAs within the gonads of teleost fish species may provide insights for the identification of markers relevant to sex development. In light of the relative lack of research on the European sea bass species in this field, delving into the European sea bass miRNome promises to offer valuable insights into the intricate relationship between the environment, miRNA expression patterns and the sexual phenotype. Thus, the aims of the present study were: (1) to enrich the knowledge of miRNA present in the European sea bass gonads; (2) to identify sex specific markers in fish; (3) to discover heat-recorders of environmental past events like temperature. To achieve our goals, miRNA-sequencing strategies of European sea bass gonads subjected to high temperatures were used. Furthermore, we undertook a comparative analysis of our European sea bass miRNA data with obtained profiles of public gonadal miRNAs databases of nine different species [32]: zebrafish, three-spined stickleback (*Gasterosteus aculeatus*), striped catfish (*Pangasianodon hypophthalmus*), Japanese medaka (*Oryzias latipes*), black bullhead (*Ameiurus melas*), European perch (*Perca fluviatilis*) and eastern mudminnow (*Umbra pygmaea*) and two Holostean species: bowfin (*Amia calva)* and spotted gar (*Lepisosteus oculatus*) [33]. This study highlights the identification of potential miRNAs as candidates for markers of heat-induced masculinization in fish gonads, as well as miRNAs exhibiting sexually dimorphic responses in the gonads across various fish species. These findings offer promising potential as tools for predicting historical environmental events linked to masculinization caused by exposure to high-temperature.

## Materials and methods

### Experimental design

The fish used in this study were siblings of those used in previous studies in which a significant male-skewed sex ratio (70.45% males to 29.55% females, *p-*value < 0.001) was observed after high temperature treatments during early development [34, 35].

One-day-post hatch (dph) European sea bass larvae were purchased from a commercial Spanish hatchery. Eggs and newly hatched larvae were kept in 19-liter cylindrical PVC containers, which were placed inside 650-liter fiberglass tanks filled with seawater. The containers were fitted with nylon mesh at the bottom, with pore sizes adjusted to match the growth stage of the larvae. Larvae were divided in two 650-l tanks and maintained at 17 °C, a temperature known to avoid temperature effects on sex ratio, for the first 20 dph. Subsequently, the temperature in one tank was gradually raised to 21 °C (high temperature, HT group), while it was lowered to 15 °C in the other (low temperature, LT group). In both cases, the temperature was adjusted at a rate of 0.5 °C per day. At 68 days post-hatching (dph), the temperature in the LT group was progressively increased to match that of the HT group, and thus all tanks returned to natural temperatures until fish achieved the sex differentiation (∼ 150–280 dpf). Around 220 dph, the temperatures of both groups were allowed to follow natural fluctuations. Therefore, the only difference in rearing conditions between the LT and HT groups was the temperature experienced during the 20–68 dph period. At 400 dpf, fish were sacrificed by an overdose of benzocaine, and biometric data were obtained. Gonads were dissected and quickly flash frozen in liquid nitrogen and kept at -80 °C.

European sea bass larvae were divided into four groups: female control temperature (FCT), female high temperature (FHT), male control temperature (MCT), and male high temperature (MHT), in duplicated tanks.

Fish were reared under daily supervision of the following environmental conditions: pH (∼ 7.9), salinity (∼ 37.8 ppt), oxygen saturation (85–100%) and with a water renewal rate of 30% vol·h − 1. The rearing density was maintained at a low level to prevent any potential impact on sex ratios, therefore it was kept at safe ranges during all developmental stages: eggs (6000–10,000 eggs·l − 1), larvae (200–350 larvae·l − 1), juveniles (10–20 fish·l − 1) and adults (11–12 kg·m − 3) (details can be found in [36]). Larvae were manually fed three times a day, starting with Artemia AF, followed by amino acid-enriched Artemia EG (INVE Aquaculture, Belgium), and then transitioning to dry food (ProAqua, S.A., Spain) of appropriate pellet sizes as the fish grew. Juveniles were fed *ad libitum* twice a day.

A total of 14 fish were selected: *N* = 8 males (four fish per treatment, two for each duplicated tank), *N* = 6 females (three fish per treatment, two from one duplicated tank and one from the other tank). In order to be able to study the heat effects uniquely, we checked the selected fish not to present biometric differences between study groups within the same sex [34, 35]; FCT: 151 g ± 25 g, 21 cm ± 1.29 cm; FHT: 169 g ± 28 g, 22 cm ± 0.95 cm; MCT 77 g ± 17.2 g, 17 cm ± 1.19 cm; MHT: 79 g ± 9 g, 17 cm ± 1 cm, *p-*values > 0.05). Animals were sacrificed via an overdose of phenoxyethanol, followed by spinal cord dislocation.

### miRNA extraction

miRNAs of 14 gonads (eight testes and six ovaries) were obtained by total mRNA by using miRNAs isolation commercial kit (Qiagen miRNA, 217004) and RNeasy MinElute Cleanup Kit to purify high quality miRNAs (Qiagen miRNA, 74204). Quality of the samples was assessed by BioAnalyzer (2100 Bioanalyzer, Agilent Technologies) with ratio 260/280 = 2.07 ± 0.04 and 260/230 = 1,73 ± 0,24) and by the RNA Integrative Number (RIN) measured with a Bioanalyzer (Agilent Technologies, USA) with values > 9.2 in testis. In ovary, RIN numbers were not considered due to naturally high levels of 5s rRNA and tRNA [37, 38].

### miRNA sequencing

In total, 14 libraries were constructed from European sea bass gonad samples. Library preparation was performed by NEBNext Small RNA Library Prep Set for Illumina (Multiplex Compatible) kit following manufacture instructions, using sequencing Lane (1 × 50, v4, HiSeq) single-end mode with a read length of 50 bp at the Genomics Unit of the Center for Genomic Regulation (CRG) service in Barcelona.

### Bioinformatics and statistical analysis

Alignment was done with Prost [39]! using the *Dicentrarchus labrax* genome assembly (version: dlabrax2021), and *Oryzias latipes* was used as reference genome for annotating the miRNAs. For the annotation, three mismatches were allowed. miRNAs without annotation were searched in the FishmiRNA database and complemented [32]. Raw reads were normalized using the DESeq2 package in R and hierarchical clustering of the sample groups was determined by a Principal Component Analysis (PCA) using the plotPCA library in the DESeq2 package [40]. Differential expression (DE) was determined by the DESeq2 package in R software and DE was considered when a minimum of 10 samples presented count reads. miRNAs with less than 10 reads in a sample were discarded. Significant differentially expressed miRNAs (DE-miRNAs) were identified using an *adjusted p*-value (*adj p*-value) cutoff of < 0.05, with logFC = 0. The adjusted *p*-value was calculated using the “results” function on the dataset obtained by the DESeqDataSetFromMatrix function, which was included in the differential expression analysis. Visualization of differential expression was accomplished by a heatmap using the pheatmap package (version 1.0.12) in R. Target genes of DEM were determined using TargetScan (v. 6.0).

### Quantitative PCR validation for miRNA-seq validation

Validation of the miRNA sequencing data was done by quantitative PCR (qPCR) of twelve selected sequenced miRNAs in all the samples used by RNA-seq. qPCR product lengths were 18–22 nucleotides and the efficiencies of the primers of the twelve selected miRNA were between 98 and 110%. A gradient qPCR was used to determine the correct annealing temperature for each primer. cDNA was generated using the miRNA 1st-Strand cDNA Synthesis Kit (Agilent Technologies) following manufacturer’s instructions. Firstly, cDNA synthesis was performed by the polyadenylation reaction. qPCR was performed using the qPCRBio SyGreen Blue Mix low ROX (PCR Biosystems). A mix of 5 µL 2x qPCRBIO SyGreen Blue mix, 0.5 µL forward primer, 0.5 µL universal reverse primer (Agilent Technologies), 2 µL of cDNA and up to 10 µL of water was prepared for each sample. The amplification program was as follows: 95 °C for 2 min, 95 °C for 10 min, followed by 40 cycles of 95 °C for 5 s and 60 to 65 °C for 20–30 s. qPCRs were run in triplicate in optically clear 384-well plates (CFX-386, Touch BioRad). Melting curve analysis was done at the end of each PCR program to ensure the uniqueness of the product.

For real-time PCR analysis, the comparative threshold cycle approach (2–ΔΔCT) was used to examine the expression level of miRNAs, followed by a Student’s t-test [41, 42]. To test validation, qPCR data and miRNA-seq data were analyzed by Pearson correlation analysis.

The sequences of the forward primers for the selected miRNAs were as follows: ola-miR-146a-3p: ATCTATGGGCTCAGTTCTTTTG, ola-miR-7132b-3p: TGAGGCGTTTAGAACAAGTTCA, ola-miR-143-3p: TGAGATGAAGCACTGTAGCTC, ola-miR-21-5p: TAGCTTATCAGACTGGTGTTGG, ola-miR-26a-2/3-b-5p: TTCAAGTAATCCAGGATAGGCT, ola-miR-199a-5p: CCCAGTGTTCAGACTACCTGTTC, ola-miR-199a-3p: ACAGTAGTCTGCACATTGGTT, ola-223-3p: TGTCAGTTTGTCAAATACCCCA, ola-192-5p: ATGACCTATGAATTGACAGCC, ola-miR-194a-5p: TGTAACAGCAACTCCATGTGGA, ola-miR-726-5p: GGAATTCCGCTAGTTCTGAACT, ola-miR-726-3p: TTCACTACTAGCAGAACTCGG. The small nuclear RNA dre-U6: ACTAAAATTGGAACGATACAGAGA was used as the reference gene.

### Conservation analysis

Expression data from miRNAs in ovary and testis were downloaded from the nine fish species in which gonadal miRNAs data were available from FishmiRNA [32], which represent a wide diversity of reproduction systems: domesticated zebrafish —PSD system— [43], three-spined stickleback—XX/XY chromosomal system— [39, 44], bowfin — XX/XY or ZZ/ZW chromosomal system — [45], striped catfish —XX/XY chromosomal system— [46] (NCBI SRA: PRJNA256963), Japanese medaka —XX/XY chromosomal system, dmrt1Y being the sex determining gene— [47, 48], spotted gar —gonochoristic— [49, 50], black bullhead —gonochoristic— [51] (NCBI SRA: PRJNA730692), European perch —XX/XY chromosomal system— [45, 52] and eastern mudminnow —gonochoristic— [45, 53].

Seed region reads from all the gathered datasets were filtered using a threshold of ≥ 1 count per million (CPM). Common miRNA seed regions between species were visualized in an upset plot using the UpSetR package (version 1.4.0). To identify highly expressed miRNA seeds in the ten studied fish species, the relative abundance was shown by calculating the percentage of miRNA abundance in the whole miRNome of the tissue, either ovary or testis. The top conserved miRNA seeds were visualized in a dotplot using the ggplot2 package (version 3.4.2). Sex-specific overexpression of mature miRNAs was determined by calculating the testis normalized read count divided by the ovary normalized read count per species. When a miRNA was not expressed in ovary or testis, a value of 1 was assigned to avoid division by 0. Afterwards, the log2 ratio>|1| was calculated for all values. Expression of conserved main sequences or mature miRNA names was considered conserved when complying to a threshold of log2ratio≥|1| in at least five species, i.e., 50% of the studied cases, among which presence in *D. labrax* was mandatory. To show the evolutionary relation between the teleost species used in the present study, a phylogenetic tree was generated using PhyloT (v2) website tool.

## Results

### miRNA sequencing and annotation

On average, 10.3 and 6.7 million sequences per library were obtained in testes and ovaries, respectively. The total number of sequences exceeded 146 million; ∼93 and ∼ 54 million for testes and ovaries, respectively. A total of 298 mature miRNAs were identified after the alignment to the *Dicentrarchus labrax* genome (version dlabrax2021) and after performing the annotation by using the *Oryzias latipes* genome (version ASM223467v1) (Dataset 1). IsomiRs, identified by Prost!, resulted in 31 out of 298 miRNAs (Dataset 1). Ten mature miRNAs were not aligned to the genome but were identified using FishmiRNA [32] and manually annotated. Thus 100% of the obtained sequences were annotated.

### miRNA-seq validation

To validate the miRNA-seq data, qPCR analysis was performed on the ovaries and testes samples of twelve selected sequenced miRNAs in all the samples used by RNA-seq. When comparing the log2FC as obtained by qPCR and miRNA-seq for twelve miRNAs, we obtained an R^2^ = 0.9426 and *p*-value = 1.6e-07 (Suppl Fig. 4), thus validating our data.

### Sexual dimorphisms in the MiRNA expression

**Fig. 1:**
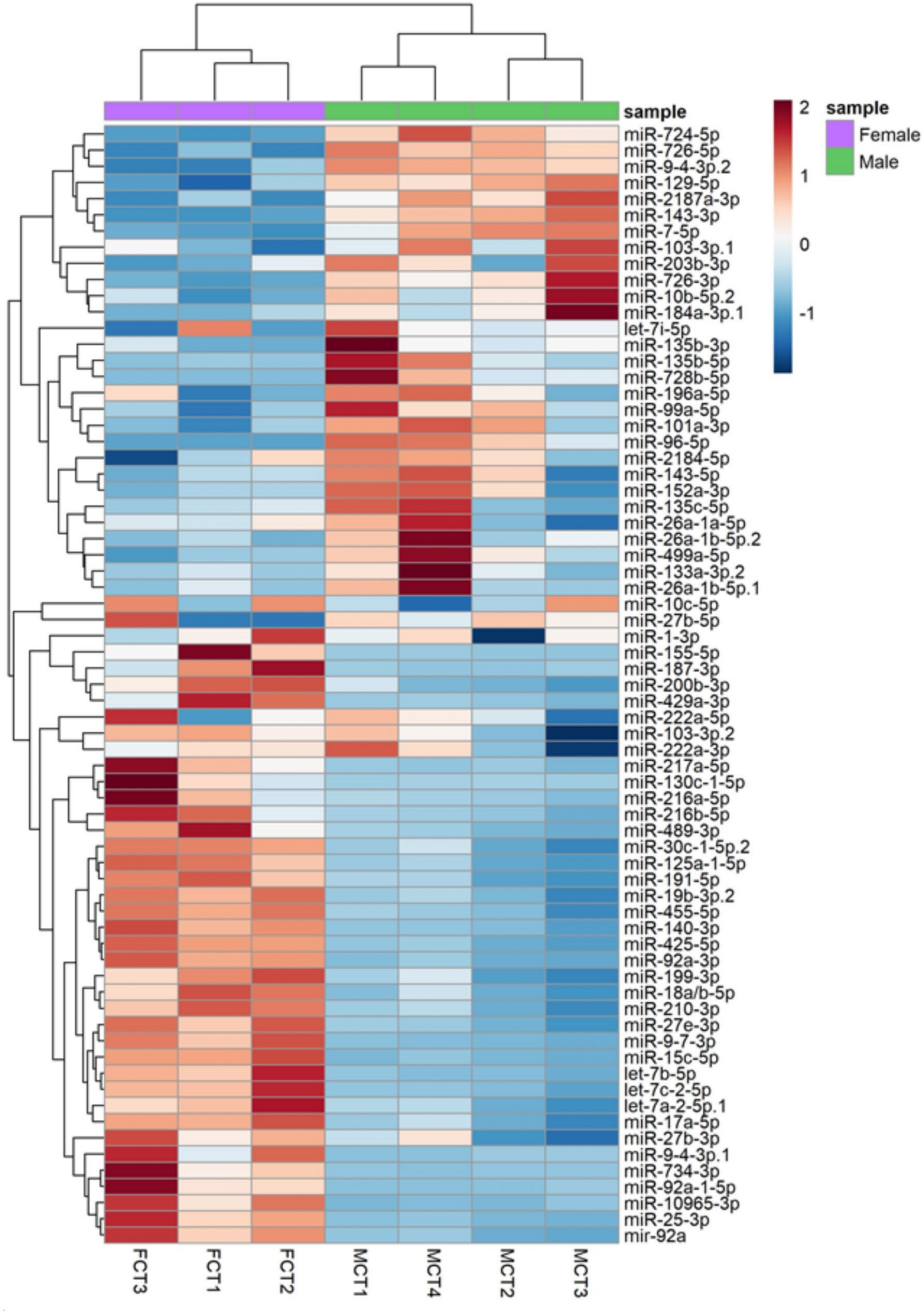
Heatmap of 69 differentially expressed miRNAs (DEM) in European sea bass (*Dicentrarchus labrax*) testes in comparison to ovaries (MCT vs. FCT). The color scale ranges from red to blue, which red shows relative upregulation in testis and blue shows relative upregulation in ovary. Both DEM and samples (*N* = 7; *N* = 4 males and *N* = 3 females) were grouped by hierarchical clustering based on sex

PCA analysis separated the two sexes, independently of the treatment, achieving a percentage of variance explained of 58% (Suppl Fig. 1A). When comparing testis vs. ovary, 69 miRNAs were found to be DE in European sea bass (*adj p-value* < 0.05), of which 33 were upregulated in ovary and 36 were upregulated in testis (Fig. 1, Dataset 2). The five most upregulated miRNAs in ovary were miR-734-3p, miR-130c-1-5p, miR-187-3p, miR-155-5p and miR-9-4-3p while in testis were miR-726-3p, miR-724-5p, miR-135b-5p, miR-184a-3p, and miR-726-5p.

### Heat sensitive MiRNAs

FHT vs. FCT and MHT vs. MCT miRNA expression clustering was shown in a PCA (Suppl Fig. 1B and C). The two components together explained 57% and 69% of the variance in ovary and testis, respectively.

**Fig. 2:**
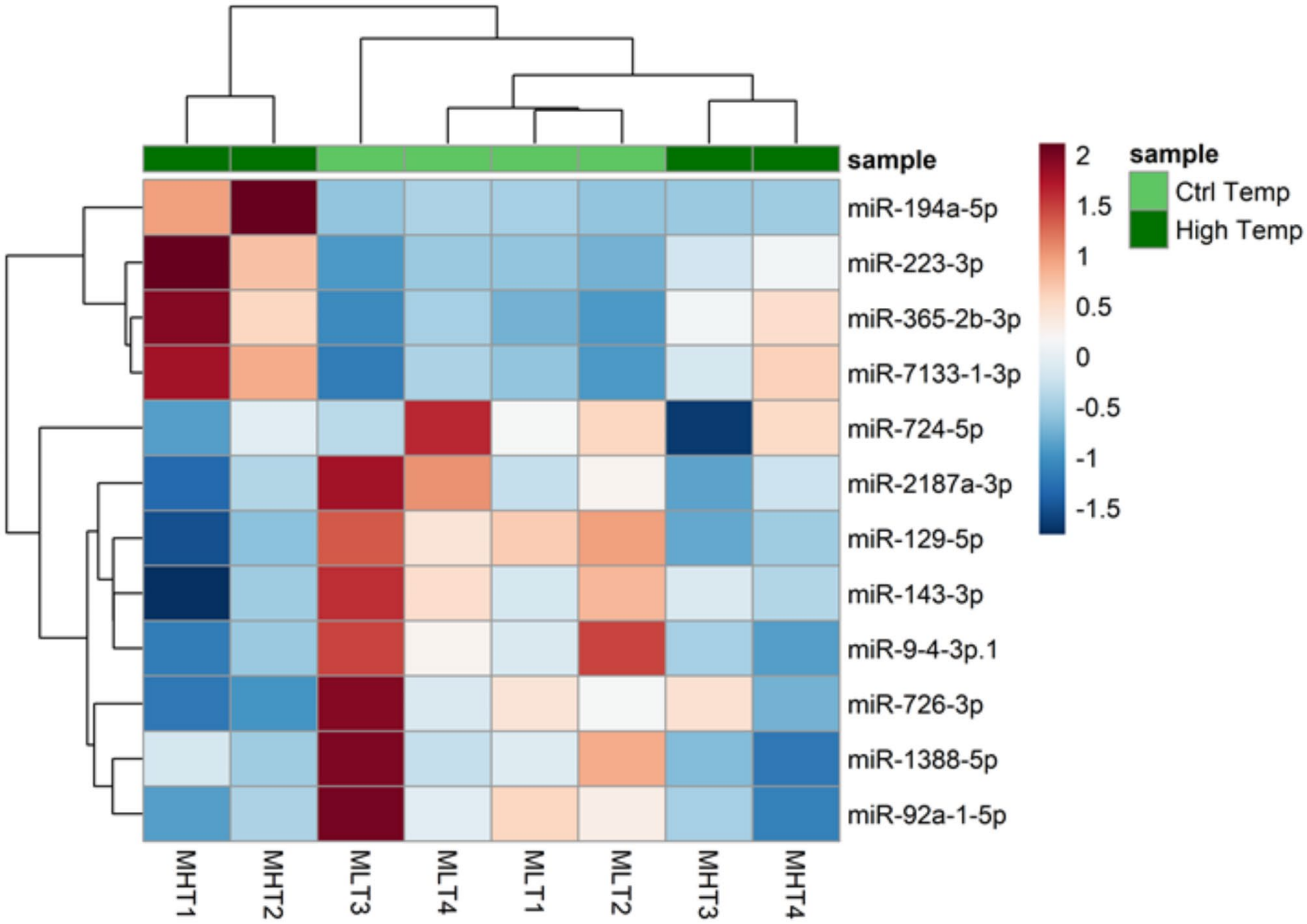
Heatmap of 12 differentially expressed miRNAs (DEM) in European sea bass (*Dicentrarchus labrax*) differentiated testes one year after heat exposure. The color scale ranges from red to blue, where red shows relative upregulation in male high temperature (MHT) males and blue is relative upregulation in male control temperature (MCT). The cutoff values were, for adjusted *p*-vales < 0.05 and log2 FC = 0

Almost one year after the end of the heat exposure period development, three miRNAs remained DE (*adj p*-value < 0.05) in ovary, one upregulated, miR-192-5p, and two downregulated, miR-143-3p and miR-146a-3p (Dataset 2). In testis, twelve DE miRNAs (DEM) were identified (*adj p*-value < 0.05, Fig. 2, Dataset 2), of which four (e.g., miR-194a and miR-223-3p) and eight (e.g., miR-92a-5p, miR-726-3p, miR-724-5p, miR-143-3p, miR-129-5p) miRNAs were upregulated and downregulated, respectively. Based on the expression patterns of the DEMs shown in the heatmap (Fig. 2), the MHT fish clustered into two distinct groups: MHT1 and MHT2 grouped separately from MHT3 and MHT4, which clustered with the control males. This clustering suggests that MHT1 and MHT2 were likely genetic females that might have been masculinized due to heat treatments.

Reproduction-related genes were predicted as target genes of male DEM (Dataset 3), such as *tdrd6* (miR-1388-5p, miR-194a-5p, miR-2187a-3p, miR-9-4-3p and miR-92a-1-5p), *sox3* (miR-129-5p, miR-223-3p, miR-7133-3p), *pgrmc1* (miR-365-2b-3p, miR-7133-3p, miR-724-5p) and *gper1* (miR-129-5p, miR-1388-5p, miR-2187a-3p, miR-7133-1-3p, miR-724-5p, miR-9-4-3p, miR-92a-1-5p). Supplementary Figure S2 shows the number of predicted target genes for each of the identified miRNAs in the Reproduction process GO term.

### miRNA conservation

**Fig. 3:**
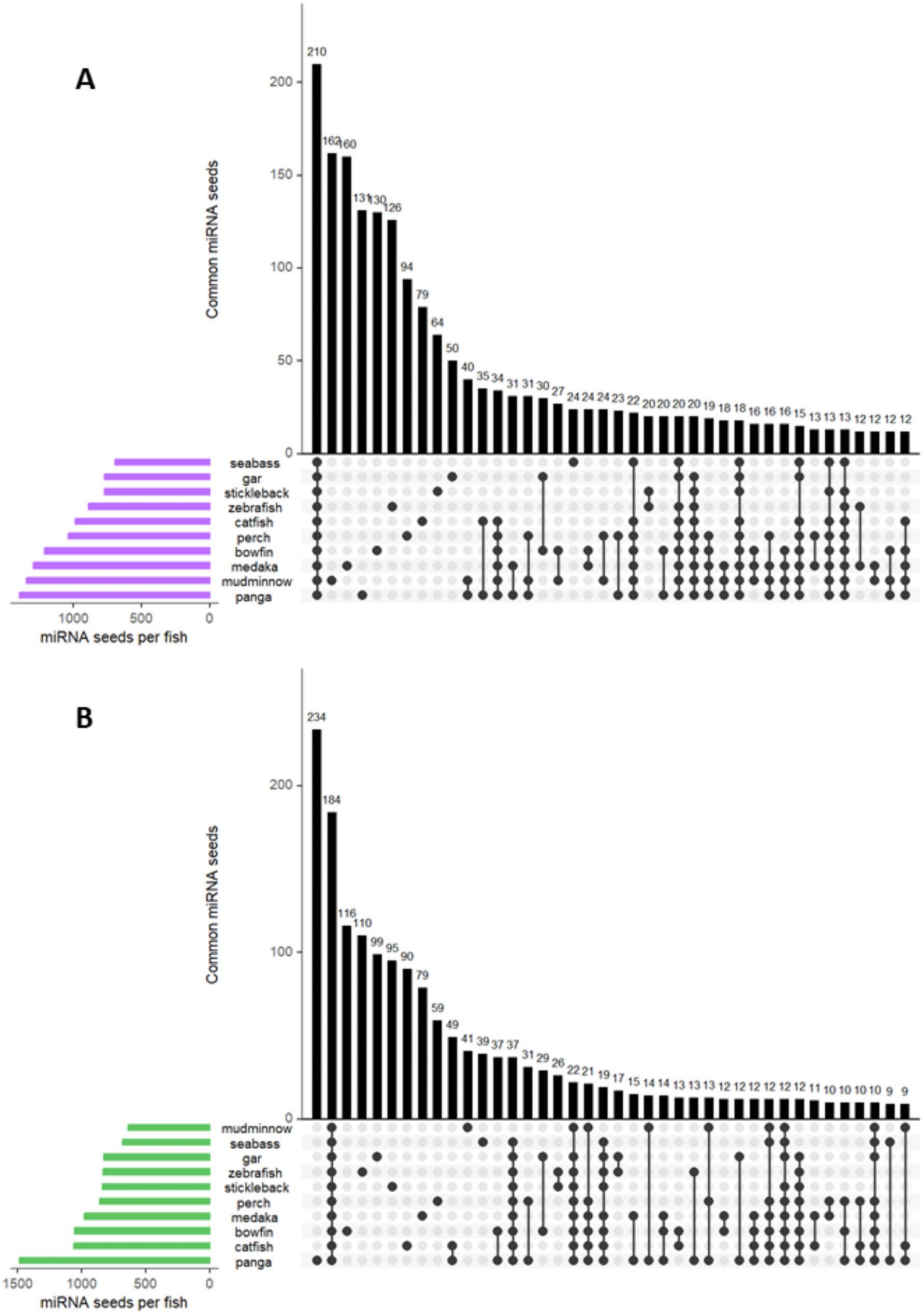
Upset plot of conserved seed regions in ovary (**A**) and testis (**B**) in ten teleost species analyzed: *Danio rerio*, *Dicentrarchus labrax*, *Gasterosteus aculeatus*, *Amia calva*, *Pangasianodon hypophthalmus*, *Oryzias latipes*, *Lepisosteus oculatus*, *Ameiurus melas*, *Perca fluviatilis* and *Umbra pygmaea*. In ovary (green), 210 seed region sequences were conserved in all ten species. In testis (purple), 184 seed region sequences were conserved in all ten species

In order to determine conserved expression across evolution in many fish species, miRNA gonadal data from nine fish species were used together with that of European sea bass of the present study. By using an upset plot, data showed three intersecting sets (Fig. 3, Dataset 4): (1) the number of common seed region sequences between the ten species; (2) those seeds that were conserved in less than ten species and (3) the miRNA seeds per each species. In total, 210 seed regions belonging to 105 miRNAs in ovaries (Fig. 3A) and in testes 184 seed region sequences belonging to 98 miRNAs were expressed in all ten species (Fig. 3B). The evolutionary distances between species are shown in a phylogenetic tree in Suppl Fig. 3 made up of two clades of the studied species Holostei and Teleostei, emerged ∼ 250 and ∼ 310 million years, respectively.

### Abundancy of gonadal MiRNAs expression across evolution

**Fig. 4:**
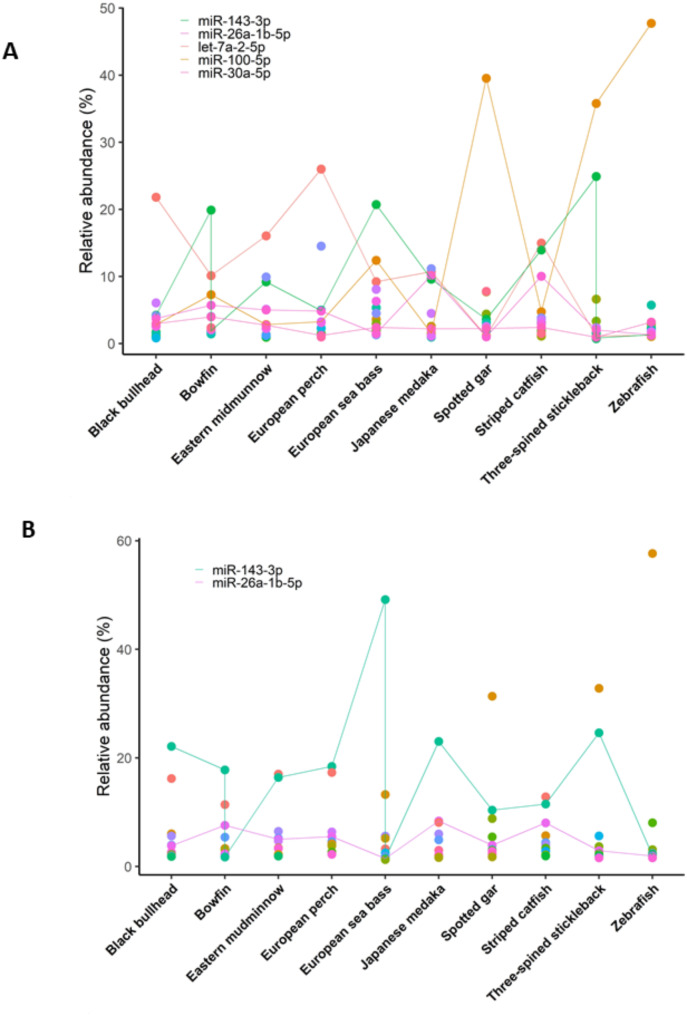
Dot plot of the top 10 most abundant miRNAs in ovary (**A**) or testis (**B**) of ten different teleost species. miRNAs expressed in all ten species are represented by a continuous line. The other miRNAs that were in the top 10 most abundant miRNAs in the ovary or testis were plotted as separated dots in different colors. The 10 most abundant miRNAs per species in ovary and testis can be found in Dataset 5

To identify highly expressed miRNA patterns throughout evolution in fish, the relative abundance of the top 10 most expressed miRNAs was calculated (Fig. 4). In ovary, five miRNAs were highly conserved: miR-143-3p, miR-26a-5p, let-7a-5p miR-100-5p and miR-30a-5p in all ten species (Fig. 4A) whereas in testis, two miRNAs, namely miR-143-3p and miR-26a-5p, were identified in all ten species (Fig. 4B). miRNAs expressed in all ten species are represented by dots connected in a continuous line, while the other miRNAs, which were among the top 10 most abundant in the ovary or testis, are plotted as separated dots in various colors. The ten highest expressed miRNAs per species in ovary and testis can be found in Dataset 5.

### miRNA gonadal markers

To identify sex-skewed miRNA expression in different fish species and miRNAs markers as heat recorders, the following analyses were performed. First, miRNAs were identified with a log2 ratio>|1| of testis vs. ovary in at least five or more species resulting a total of 33 miRNAs (Table 1). From those 21 identified as female-biased miRNA markers and 12 as male-biased markers because they were more expressed in either ovaries or testes in at least five species across evolution.

**Table 1:** Sex-skewed miRNA expression in different fish species and the effects of heat treatment. MiRNAs were identified with log2 ratio>|1| of seed region expression in testis vs. ovary in at least five or more fish species, among which European sea bass. Furthermore, each miRNA was significantly differential expressed (DEM, *adjusted-p* value < 0.05) in our data. European sea bass DEM comparisons were: (1) male high temperature (MHT) vs. male control temperature (MCT); (2) female high temperature (FHT) vs. female control temperature (FCT); (3) MCT *vs*. FCT; (4) MHT *vs*. FHT. Negative values mean the upregulation in ovary whereas positive values mean upregulation in testes for each species. Data in the table was organized based on the MiRNA conservation in the ovaries or testes along fish evolution. The DEM column shows the comparisons (1, 2, 3, and/or 4) in which each miRNA was differentially expressed for the European sea bass data. ‘count O’ and ‘count T’ represent the number of times the miRNA was differentially expressed in the ovaries or testes, respectively, in the studied species. The miRNA name corresponds to the seed region annotation aligned with European sea bass (*Dincentrarcus labrax*). Abbreviations: Dla: *Dicentrarchus labrax*; Ola: *Oryzias latipes*; Aca: *Amia calva*; Gac: *Gasterosteus aculeatus*; Dre: *Danio rerio*; Loc: *Lepisosteus oculatus*; Phy: *Pangasianodon hypophthalmus*; Pfl: *Perca fluviatilis*; Ame: *Ameiurus melas*; Upy: *Umbra pygmaea; O: ovaries; T: testes*

miRNA	Sex	dla	ola	aca	gac	dre	loc	phy	pfl	ame	upy	DEM	Count O	Count T
miR-140-3p	♀	-1,72	-1,14	-1,01	1,37		-2,43		-1,16	1,19	-2,8	3 + 4	5	2
miR-429a-3p	♀	-3,05	-2,81	-3,05		-2,5	-2,86			1,15		3 + 4	5	1
miR-140-5p	♀	-2		-1,55		-1,63	-1,23		-2,27		-2,36	3 + 4	5	0
miR-191-5p	♀	-2,05	-2,07				-1,18	-1,29	-1,94			3 + 4	5	0
miR-425-5p	♀	-2,88	-2				-1,12	-1,31	-2,82			3 + 4	5	0
miR-155-5p	♀	-4,15	-3,82	-1,18				-1,59	-4,26			3 + 4	5	0
miR-223-3p	♀	-1,11	-4,16	-1,1		-1,8			-1,56			1 + 4	5	0
miR-142-3p	♀	-1,17	-1,22	-1,97		-2,99	1,86		-4,3			4	5	1
miR-34a-5p	♀	-2,52				-2,2	-1,7	1,52	2,12	-2,28	-1,59	4	4	2
miR-148a-3p	♀	-1,28	-1,45	-2,45		-2,15		1,19	-1,03		-1,45	3	5	1
miR-200b-3p	♀	-1,88	-2,51		3,6	-2,86			-2,74		-2,47	3	4	1
miR-146a-5p	♀	-1,08	-1,15	-1,65	1,29	-1,72	-2,01		-2,79			2	6	1
miR-192-5p	♀	-2,06	-1,45	-1,32	4,91			-2,99	2,38	3,71	-2,53	2	4	3
miR-200a-3p	♀	-1,48	-2,59	-3,97	3,8	-2,7	-2,27		-3,28		-3,65		6	1
miR-27a-3p	♀	-1,11	-2,72		1,34	-1,23	1,26	-1,37	-1,56	1,68			5	3
miR-7132b-5p	♀	-1,37	-1,23		-1,6			-2,26	-4,43	2,3	1,29		5	2
miR-200b-5p	♀	-3,01	-3,38		3,16	-3,7			-4,04	-3,13	-5,36		5	1
miR-1388-3p	♀	-1,4	-1,93	-2,06		-1,66			-1,51		-5,81		5	0
miR-33-5p	♀	-1,47	-1,44	-2	1,06		2,74		3,81	-1,19	-2,45		4	3
miR-223-5p	♀	-1,91	-3,59			-2,4	1,54		-7,07	3,75	-3,77		4	2
miR-193a-2-5p	♀	-1,99	-2,83		-2,16				-4,45		-5,97		4	0
miR-143-3p	♂	1,25	1,25				1,53		1,91	2,38		1 + 2 + 3 + 4	0	5
miR-129-5p	♂	2,65	1,93	2,99			2,21		1,36	4,27	7,82	1 + 3 + 4	0	7
miR-724-5p	♂	3,85		-1,98	5,4		1,83	1,1		-3,12	4,74	1 + 3 + 4	2	5
miR-499a-5p	♂	2,23	-3,3		1,29	3,96	1,95	-1,98	2,48	2,28	3,51	3 + 4	2	7
miR-135b-5p	♂	3,76	1,79		3,34	1,69	-3,76	3	5,13		4,25	3 + 4	1	7
miR-182a-5p	♂	1,84	-1,19		2,26	3,7		-3,06	3,81	2		3 + 4	2	5
miR-7-5p	♂	2,85	4,36		-3,05	1,14			1,88	3,82		3 + 4	1	5
miR-135b-3p	♂	2,76	2,02		2,14		-2,99	1,06			5,19	3 + 4	1	5
miR-132b-5p	♂	2,11		2,29	1,89	1,09		-2,39		-1,9	6,19	3	2	5
miR-2187a-5p	♂	3,44	1,59			2,32			1,94		3,61	4	0	5
miR-212a-5p	♂	2,12		3,04	-1,24	3,18		-3,36	2,81	3,39	-6,72		2	5
miR-15c-3p	♂	1,93	-2,38		1,57		2,6	2,86		2,29	-2,77		1	5

### miRNA markers for temperature-induced sex-reversal

**Fig. 5:**
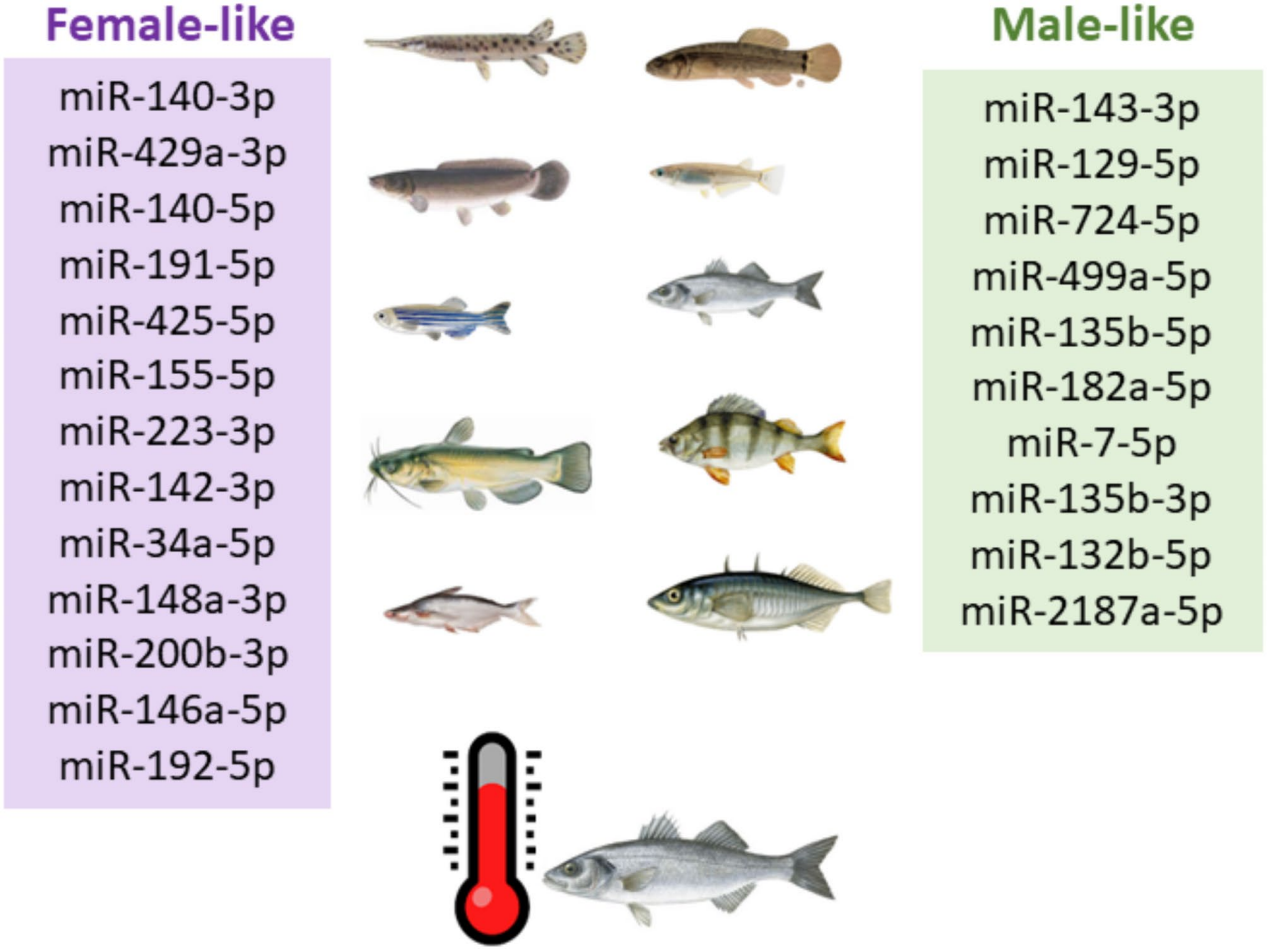
Gonadal miRNAs markers identified in this study having had across evolution. miRNAs were filtered based on the heat-treated differential expressed (DEM, *adjusted-p* value < 0.05) data obtained in European sea bass, in the different comparisons based on sex and heat treatment. miRNAs were identified with log2 ratio>|1| of seed region expression in testis vs. ovary in at least five or more fish species, among which European sea bass. Furthermore, each miRNA was significantly differential expressed (DEM, *adjusted-p* value < 0.05) in our data. European sea bass DEM comparisons were: (1) Male high temperature (MHT) vs. Male control temperature (MCT); (2) Female high temperature (FHT) vs. Female control temperature (FCT); (3) MCT vs. FCT; (4) MHT vs. FHT. In purple boxes, the female-like miRNAs were represented and in green, the male-like miRNAs with log2 ratio>|1| of seed region expression in testis vs. ovary in at least five or more fish species

miRNAs were further filtered based on the heat-treated data obtained in European sea bass in the different comparisons based on sex and heat treatment: (1) MHT vs. MCT, (2) FHT vs. FCT, (3) MCT *vs*. FCT, and (4) MHT vs. FHT). This resulted in a total of 23 miRNAs (Fig. 5), thirteen of which were female-like miRNA markers, for example, miR-140-3p, miR-429a-3p, miR-191-5p and miR-223-3p, and ten as male-like miRNA markers, for example, miR- miR-143-3p, miR-129-5p, miR-724-5p and 2187a-3p, which were found in at least in one of the studied comparisons. Data in Table 1 were organized based on the miRNA conservation in the ovaries or testes along fish evolution, the DEM column showing the comparisons (1, 2, 3, and/or 4) in which each miRNA was differentially expressed for the European sea bass data. Most of the species showed similar sex-skewed patterns of these miRNA markers, except *G. aculeatus* and *A. melas* species, whose expression patterns differed, in particular, from those identified as female-like miRNA markers. ‘Count O’ and ‘Count T’ represent the number of times the miRNA was differentially expressed in the ovaries or testes, respectively, in the studied species.

**Fig. 6:**
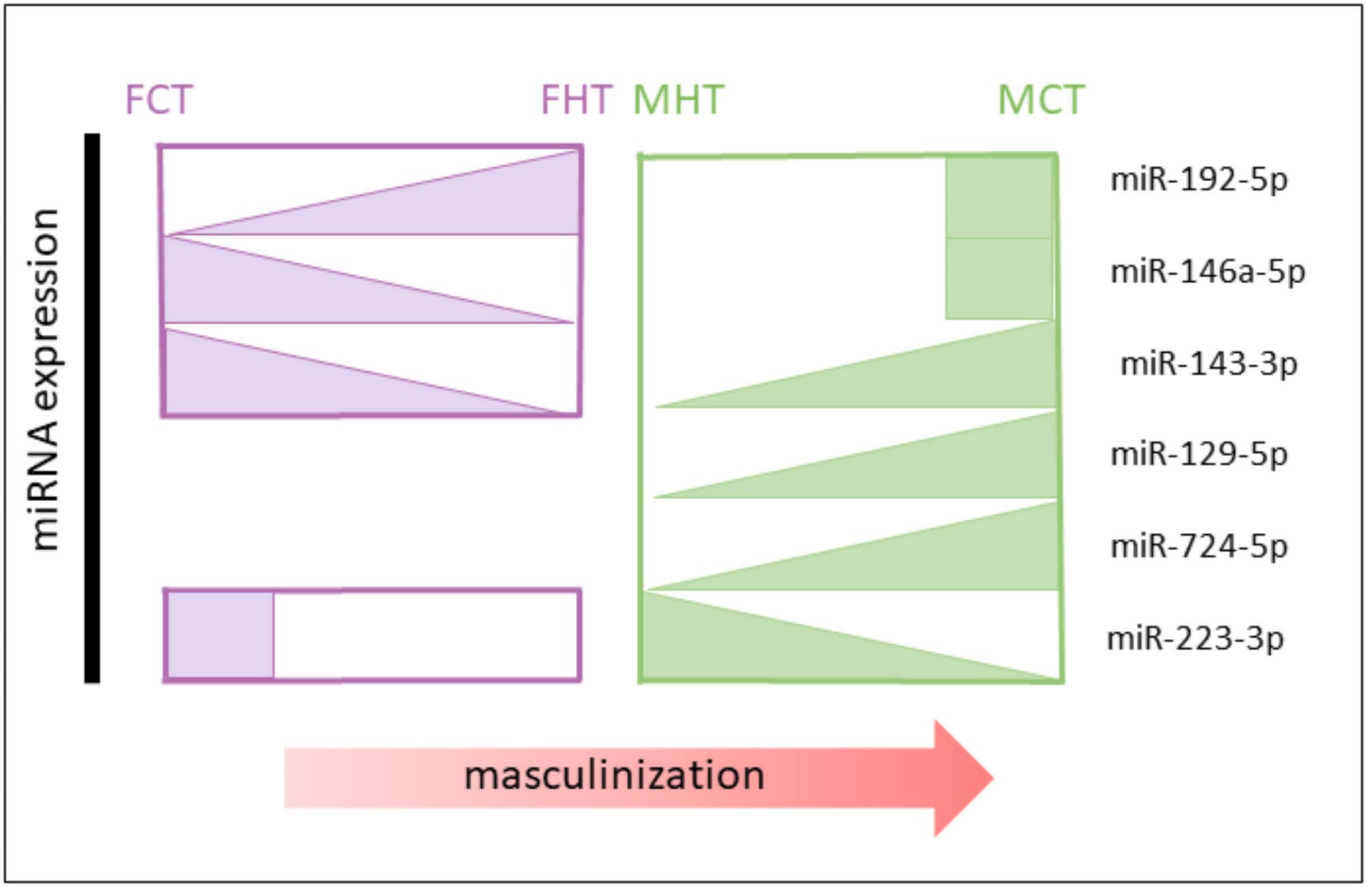
miRNA markers for temperature-induced sex-reversal proposed as heat-recorders. miRNAs were identified with log2 ratio>|1| of seed region expression in testis vs. ovary in at least five or more fish species, European sea bass among them. Furthermore, each miRNA was significantly differential expressed (DEM, adjusted-*p* value < 0.05) in our data in the DEM comparisons: (1) Male high temperature (MHT) vs. Male control temperature (MCT); (2) Female high temperature (FHT) vs. Female control temperature (FCT). miRNAs are represented by triangles. Boxes show expression of the miRNA in the ovaries or testes in control groups

For the comparison (2; FHT vs. FCT), two miRNAs were altered in the heat-treated ovaries, miR-192-5p and miR-146a-5p, which were upregulated and identified as female-like markers. Further, miR-143-3p was downregulated in ovaries after the treatment, but also previously identified as male-like marker. From the comparison (1; MHT vs. MCT), a total of four miRNAs altered the expression after one year of the heat treatment in males. Three of them, i.e., miR-143-3p, miR-129-5p and, miR-724-5p, were downregulated in testes subjected to HT in European sea bass but also identified as male-like miRNAs across evolution (Fig. 6).

Specifically, miR-143-3p was identified as DEM in all the four comparisons studied (1), (2), (3) and (4), indicating that this miRNA was heat sensitive in European sea bass, because it showed a downregulation in both heat-treated testes and ovaries and further, miR-143-3p was highly conserved in testes across evolution. In contrast to the other three male-skewed miRNAs identified, miR-223-3p was upregulated in European sea bass heated males but also expressed in ovaries across evolution, therefore previously identified as a female-like marker.

## Discussion

High temperatures can induce masculinization of fish populations. This phenomenon has significant implications for many farmed species, including the European sea bass, in which females grow to a larger size than males. In addition, increasing temperatures in our seas and oceans due to climate change have drastic implications in fish physiology and evolution (Alfonso et al. 2020). Here, we study the effect on the gonadal miRNome of this heat-induced masculinization and further explore the gonadal miRNome of other species in an effort to decipher conserved miRNA sex markers.

Long-term effects, ranging from days to months, of heat treatment on the miRNA expression profile in the gonads have been previously described in fish, long terms. In particular, in Atlantic cod, miR-27c, miR-30c, and miR-200a were found to be DE in juveniles after embryonic or larval heat-treatment. However, no distinction between males or females was addressed [22]. Other studies in zebrafish describing the heat stress by temperature in the gonadal miRNome revealed that miR-22b and miR-301a were altered to be fully recovered 21 days after the exposure [23], thus not being appropriate as a long-term markers. In zebrafish adult females, 23 miRNAs were DE in ovaries after larval heat-treatment, among which, for example, dre-miR-726-3p was downregulated while dre-miR-724-5p was upregulated three months after the heat treatment [21]. Interestingly, these two miRNAs were both found to be downregulated in European sea bass testis one year after heat exposure in the present study, indicating that a conservation in the heat response among species may exist, although the expression pattern might not always be conserved. Similarly, miR-143-3p and miR-92a-3p, which were downregulated in high temperature treated male fish in the present study, were also altered in mice testes exposed for only 25 min to elevated temperatures, highlighting them as potential markers in heat-induced spermatogenesis dysregulation [54]. Higher levels of circulating miR-143 and miR-223 were detected in plasma levels together with cortisol after acute stress response in European sea bass [55]. In the present study, both miRNAs (miR-143 and miR-223) were altered after heat treatment, indicating that they could be potential makers for stress response. A heat-treatment experiment with exact the same conditions in E. sea bass, no differences were observed in the presence and abundance of different cell types between high-temperature and low-temperature fish —females had immature ovaries containing oocytes at the cortical alveolar stage while males had testes containing all germ cell types, including spermatozoa, although no sperm— [35]. Thus, the differences observed in the present study were due to the heat treatment during early development, one year before we measured the alterations in the miRNome, and not due to gonadal development, sperm quality, or puberty. Overall, our data showed that in fish, miRNAs are sensitive to drastic temperature changes in the environment and could play a role in regulating thermal plasticity.

In European sea bass, we observed sexual dimorphism in miRNA expression within fish gonads, with 33 upregulated and 36 downregulated miRNAs in the ovary compared to the testis. To enhance reliability, we cross-referenced our data with existing datasets from the nine species, identifying common gonadal miRNAs across evolution. In testes and ovaries, we have identified 184 and 210 conserved seed region sequences in the ten studied fish species, respectively. Although we have detected the expression of these miRNAs in the gonads in many fish species, these miRNA seed sequences were not sex-specific or tissue-specific, as many miRNAs are highly conserved throughout evolution and body plan development. For example, miR-100 and let-7, were considered to be “ancestral miRNAs”, due to its conservation already described within Metazoan species [26, 56, 57]. Nevertheless, a conservation analysis revealed 74 conserved miRNAs divided into three groups: 23 miRNA families were present in both protostomes and deuterostomes, 46 families conserved exclusively in vertebrates, and 5 families (mir-430, mir-722, mir-724, mir-734, and mir-738) exclusively for fish species [58]. Notably, in our data, miR-143-3p, miR-26a-5p, let-7a-5p, miR-100-5p, and miR-30a-5p in the ovaries, and miR-143-3p and miR-26a-5p in the testes, were the most expressed miRNAs in all ten studied fish species. Similarly, in olive flounder (*Paralichthys olivaceus*), miR-143, miR-26a and let-7a were also identified as the most expressed miRNAs in both ovaries and testes [59]. In mouse and human, miR-30a-5p was crucial for spermatogonial stem cell differentiation [60] and was used as a marker for men suffering from non-obstructive azoospermia [61]. In our data, however, we observed a conserved overexpression of miR-30a in adult fish ovaries compared to adult testes in the ten studied species. Nevertheless, in Nile tilapia, miR-30a presented higher expression in males at 5 days after hatching (dah) and it was able to downregulate *cyp19a1a* in gonads [62]. We identified miR-26a both in ovaries and testes, while in Chinese tongue sole (*Cynoglossus semilaevis*), was identified as transgenerational male marker in sperm [63]. Overall, data showed the complexity of the presence of miRNA across evolution and their multiple roles in regulating many biological processes, even those involved in the reproductive system.

The exploration of miRNA phylogenetic conservation and diversity indicates that miRNAs play crucial roles in animal evolution by influencing phenotypic variation during development [25]. This might explain the different sexual patterns observed in the gonads between the ten fish species of two different clades (i.e., Teleostei and Holostei) separated from one species to others by millions of years of evolution. Further, the fish species used in the present study represent distinct reproduction systems, from XX/XY chromosomal system such as medaka, stickleback, perch and, panga [44, 46, 52, 64], to polygenic system such as European sea bass and domesticated zebrafish [8, 65]. Thus, to detect sexual dimorphic miRNA markers in the fish gonads, we considered a sex-biased miRNA when the expression was found in at least five studied species. In total, we identified thirteen and ten female-like and male-like markers, respectively. For example, miR-429a-3p and miR-140-3p were overexpressed in ovary in at least five studied species. miR-429b-3p was reported to have lower expression in yellow catfish in YY super males compared to XY males [66] and in chicken, miR-140-3p was shown to promote germ cell proliferation while targeting anti-Müllerian hormone *amh* [67]. Scarce data exist about miR-499, which has been related to cardiac disorders, lung cancer and regulating circadian clock [68–70] in mammals. In fish, miR-499 plays a role in muscle tissue [71–73]. Further, its evolution targeting the intronic region of the myosin heavy chain (MYH14) gene was studied throughout evolution in teleosts [74]. Recently, in the European sea bass, higher levels of circulating miR-499 in plasma differentiating males were identified, when compared to differentiating females [55]. Another example of a male-like miRNA in fish gonads is miR-135b-3p, which is a biomedical marker in humans due to its involvement in many cancer types and disorders like Alzheimer’s disease [75, 76]. Similar to our data, in tilapia, miR-135 presented sexual dimorphism towards males as it was upregulated in testes together with miR-33a, miR-132 and miR-212 [77, 78].

To identify miRNA markers that convey information about both sexual dimorphism and serve as recorders of environmental cues, we considered the sex-biased miRNAs across evolution together with the data from the heated European sea bass. These analyses aided in pinpointing miRNAs that could be accountable for the masculinizing effects induced by high temperatures, detecting a total of five informative miRNAs. miR-223-3p was upregulated in HT males but overexpressed in ovaries in at least five fish species, thus indicating that they might be involved in the masculinization of the gonads. Therefore, it might be hypothesized that the differentiated testes heated with high temperatures during early development might belong to genetic females, similarly to what has been described in heated-temperature female zebrafish presenting a male-like transcriptome [9]. Nevertheless, reliable sexual-genetic makers — which unfortunately are not available in European sea bass —would be required to further confirm the original sex of the heated animals. In Chinese sole tongue, miR-223-3p (referred to as miR-223-y) was upregulated during oocyte maturation. The expression of miR-223-3p was lower in YY super males of yellow catfish (*Pelteobagrus fulvidraco*) than XY males [66]. Further, male-like miRNAs that showed heat sensitivity were miR-129-5p, miR-724-5p and miR-143-3p, which were downregulated in HT males. In literature, miR-129 was associated with sexual maturity in rainbow trout testis [79], while miR-724-5p (referred to as miR-724-x) showed upregulation during oocyte maturation although it suppressed *cyp19a1a* [80, 81]. In mice [54], miR-143-3p was shown to be highly upregulated after heat-induced stress, reducing spermatogenesis and thus indicating the thermal sensitivity of this miRNA in the gonads. In the present study females treated with heat, miR-143-3p together with miR-146a-5p were inhibited, while miR-192-5p was upregulated due to temperature one year after the heat. In all, these identified miRNAs may serve as potential epimarkers for predicting past environmental events based on the sexual fish phenotype. The present results demonstrate that, in European sea bass, the miRNome may influence an essential biological process, with consequences for resulting population sex ratios. Although further studies are needed to confirm this, it is tempting to suggest that the miRNAs described herein may be components of the molecular mechanisms connecting environmental temperature, gonadal development, and, consequently, sex ratios.

## Conclusions

To gain a better understanding of the intricacies of masculinization induced by heat treatments during early developmental stages in European sea bass, we detected a total of three and twelve miRNAs in the ovaries and testes, respectively. To explore the sexual dimorphic miRNA patterns in the gonads across different fish species, we integrated our findings with available miRNAs from public databases. This analysis identified five evolutionarily conserved miRNAs (miR-146a-5p, miR-143-3p, miR-129-5p, miR-724-5p, and miR-223-3p) that could serve as sex markers. Additionally, all these identified miRNAs were altered following heat treatment in European sea bass, suggesting their potential use as candidates for conserved markers of heat-induced masculinization in fish gonads and across species. These findings hold promise as potential tools for predicting historical environmental events associated with masculinization due to high-temperature treatments in cultured species, but also perhaps in natural populations exposed to a climate change scenario.

## Electronic supplementary material

Below is the link to the electronic supplementary material.

**Supplementary Material 1:** Dataset 1

**Supplementary Material 2:** Dataset 2

**Supplementary Material 3:** Dataset 3

**Supplementary Material 4:** Dataset 4

**Supplementary Material 5:** Dataset 5

**Supplementary Material 6:** Supplementary Figures

## Data Availability

Raw sequencing data generated during the current study can be found in NCBI SRA repository with the accession number: PRJNA1008584. The raw data will be publicly available upon publication and the metadata can be accessed by reviewers through: https://dataview.ncbi.nlm.nih.gov/object/PRJNA1008584?reviewer=ct6r6cbgav3c2ru7evoqvlkqg7.

## References

[1] Vandeputte M, Gagnaire P-A, Allal F. The European sea bass: a key marine fish model in the wild and in aquaculture. Anim Genet. 2019;50:195–206. 30883830 10.1111/age.12779 PMC6593706

[2] FAO. FAO; 2022.

[3] Mei J, Gui J-F. Genetic basis and biotechnological manipulation of sexual dimorphism and sex determination in fish. Sci China Life Sci. 2015;58:124–36. 25563981 10.1007/s11427-014-4797-9

[4] Saillant E, Fostier A, Haffray P, Menu B, Thimonier J, Chatain B. Temperature effects and genotype-temperature interactions on sex determination in the European sea bass (*Dicentrarchus labrax L*). J Exp Zool. 2002;292:494–505. 11857484 10.1002/jez.10071

[5] Piferrer F, Blázquez M, Navarro L, González A. Genetic, endocrine, and environmental components of sex determination and differentiation in the European sea bass (*Dicentrarchus labrax L*). Gen Comp Endocrinol. 2005;142:102–10. 15862554 10.1016/j.ygcen.2005.02.011

[6] Navarro-Martín L, Blázquez M, Viñas J, Joly S, Piferrer F. Balancing the effects of rearing at low temperature during early development on sex ratios, growth and maturation in the European sea bass (Dicentrarchus labrax).: limitations and opportunities for the production of highly female-biased stocks. Aquaculture. 2009;296:347–58.

[7] Wang H-P, Shen Z-G. Sex control in aquaculture. Sex control in aquaculture. Chichester, UK: John Wiley & Sons, Ltd; 2018. pp. 1–34.

[8] Vandeputte M, Dupont-Nivet M, Chavanne H, Chatain B. A polygenic hypothesis for sex determination in the European sea bass *Dicentrarchus labrax*. Genetics. 2007;176:1049. 17435246 10.1534/genetics.107.072140 PMC1894574

[9] Ribas L, Liew WC, Díaz N, Sreenivasan R, Orbán L, Piferrer F. Heat-induced masculinization in domesticated zebrafish is family-specific & yields a set of different gonadal transcriptomes. Proc Natl Acad Sci U S A. 2017;114:E941–50. 28115725 10.1073/pnas.1609411114 PMC5307468

[10] Fernandino JI, Hattori RS, Kishii A, Strüssmann CA, Somoza GM. The cortisol and androgen pathways cross talk in high Temperature-Induced masculinization: the 11β-Hydroxysteroid dehydrogenase as a key enzyme. Endocrinology. 2012;153:6003–11. 23041673 10.1210/en.2012-1517

[11] Shang EHH, Yu RMK, Wu RSS. Hypoxia affects sex differentiation and development, leading to a Male-Dominated population in zebrafish (Danio rerio). Environ Sci Technol. 2006;40:3118–22. 16719120 10.1021/es0522579

[12] Ribas L, Valdivieso A, Díaz N, Piferrer F. Appropriate rearing density in domesticated zebrafish to avoid masculinization: links with the stress response. J Exp Biol. 2017;220:1056–64. 28082617 10.1242/jeb.144980

[13] Ribas L, Vanezis K, Imués MA, Piferrer F. Treatment with a DNA methyltransferase inhibitor feminizes zebrafish and induces long-term expression changes in the gonads. Epigenetics Chromatin. 2017;10. 10.1186/s13072-017-0168-7 PMC5721477 29216900

[14] Moraleda-Prados J, Caballero-Huertas M, Valdivieso A, Joly S, Ji J, Roher N, et al. Epigenetic differences in the innate response after immune stimulation during zebrafish sex differentiation. Dev Comp Immunol. 2021;114:103848. 32888969 10.1016/j.dci.2020.103848

[15] Granada L, Lemos MFL, Cabral HN, Bossier P, Novais SC. Epigenetics in aquaculture – the last frontier. Rev Aquac. 2018;10:994–1013.

[16] Navarro-Martín L, Viñas J, Ribas L, Díaz N, Gutiérrez A, Di Croce L, et al. DNA methylation of the gonadal aromatase (cyp19a) promoter is involved in Temperature-Dependent sex ratio shifts in the European sea bass. PLoS Genet. 2011;7:e1002447. 22242011 10.1371/journal.pgen.1002447 PMC3248465

[17] Zhou H, Zhuang ZX, Sun YQ, Chen Q, Zheng XY, Liang YT et al. Changes in DNA methylation during epigenetic-associated sex reversal under low temperature in Takifugu rubripes. PLoS ONE. 2019;14. 10.1371/journal.pone.0221641 PMC6711519 31454376

[18] Shao C, Li Q, Chen S, Zhang P, Lian J, Hu Q, et al. Epigenetic modification and inheritance in sexual reversal of fish. Genome Res. 2014;24:604–15. 24487721 10.1101/gr.162172.113 PMC3975060

[19] Sun JL, Zhao LL, Wu H, Lian WQ, Cui C, Du ZJ, et al. Analysis of miRNA-seq in the liver of common carp (Cyprinus Carpio L.) in response to different environmental temperatures. Funct Integr Genomics. 2019;19:265–80. 30443850 10.1007/s10142-018-0643-7

[20] Vasadia DJ, Zippay ML, Place SP. Characterization of thermally sensitive MiRNAs reveals a central role of the FoxO signaling pathway in regulating the cellular stress response of an extreme stenotherm, trematomus bernacchii. Mar Genomics. 2019;48:100698. 31307923 10.1016/j.margen.2019.100698

[21] van Gelderen TA, Montfort J, Álvarez-Dios JA, Thermes V, Piferrer F, Bobe J, et al. Deciphering sex-specific MiRNAs as heat-recorders in zebrafish. Sci Rep 2022 121. 2022;12:1–14. 10.1038/s41598-022-21864-3 PMC9636255 36333360

[22] Bizuayehu TT, Johansen SD, Puvanendran V, Toften H, Babiak I. Temperature during early development has long-term effects on MicroRNA expression in Atlantic Cod. BMC Genomics. 2015;16:305. 25881242 10.1186/s12864-015-1503-7 PMC4403832

[23] Ikert H, Craig PM. Chronic exposure to Venlafaxine and increased water temperature reversibly alters MicroRNA in zebrafish gonads (*Danio rerio*). Comp Biochem Physiol - Part D Genomics Proteom. 2020;33:100634. 10.1016/j.cbd.2019.100634 31715506

[24] Papadaki M, Kaitetzidou E, Papadakis IE, Sfakianakis DG, Papandroulakis N, Mylonas CC et al. Temperature-Biased miRNA Expression Patterns during European Sea Bass (*Dicentrarchus labrax*) Development. Int J Mol Sci. 2022, Vol 23, Page 11164. 2022;23:11164. 10.3390/ijms231911164 PMC9570215 36232462

[25] Niwa R, Slack FJ. The evolution of animal MicroRNA function. Curr Opin Genet Dev. 2007;17:145–50. 17317150 10.1016/j.gde.2007.02.004

[26] Christodoulou F, Raible F, Tomer R, Simakov O, Trachana K, Klaus S, et al. Ancient animal MicroRNAs and the evolution of tissue identity. Nat 2010 4637284. 2010;463:1084–8. 10.1038/nature08744 PMC2981144 20118916

[27] Hamam R, Hamam D, Alsaleh KA, Kassem M, Zaher W, Alfayez M, et al. Circulating MicroRNAs in breast cancer: novel diagnostic and prognostic biomarkers. Cell Death Dis. 2017;8:e3045–3045. 28880270 10.1038/cddis.2017.440 PMC5636984

[28] Lim LP, Glasner ME, Yekta S, Burge CB, Bartel DP. Vertebrate MicroRNA genes. Science. 2003;299:1540. 12624257 10.1126/science.1080372

[29] Cardona E, Guyomar C, Desvignes T, Montfort J, Guendouz S, Postlethwait JH, et al. Circulating MiRNA repertoire as a biomarker of metabolic and reproductive States in rainbow trout. BMC Biol. 2021;19:235. 34781956 10.1186/s12915-021-01163-5 PMC8594080

[30] Huang CW, Li YH, Hu SY, Chi JR, Lin GH, Lin CC, et al. Differential expression patterns of growth-related MicroRNAs in the skeletal muscle of nile tilapia (*Oreochromis niloticus*). J Anim Sci. 2012;90:4266–79. 22745188 10.2527/jas.2012-5142

[31] Blödorn EB, Domingues WB, Nunes LS, Komninou ER, Pinhal D, Campos VF. MicroRNA roles and their potential use as selection tool to cold tolerance of domesticated teleostean species: A systematic review. Aquaculture. 2021;540:736747.

[32] Desvignes T, Bardou P, Montfort J, Sydes J, Guyomar C, George S et al. FishmiRNA: an evolutionarily supported MicroRNA annotation and expression database for Ray-Finned fishes. Mol Biol Evol. 2022;39. 10.1093/molbev/msac004 PMC8826519 35020925

[33] Desvignes T, Carey A, Postlethwait JH. Evolution of caudal fin ray development and caudal fin hypural diastema complex in spotted Gar, teleosts, and other neopterygian fishes. Dev Dyn. 2018;247:832–53. 29569346 10.1002/dvdy.24630 PMC5980753

[34] Díaz N, Piferrer F. Estrogen exposure overrides the masculinizing effect of elevated temperature by a downregulation of the key genes implicated in sexual differentiation in a fish with mixed genetic and environmental sex determination. BMC Genomics. 2017;18:973. 29254503 10.1186/s12864-017-4345-7 PMC5735924

[35] Díaz N, Piferrer F. Lasting effects of early exposure to temperature on the gonadal transcriptome at the time of sex differentiation in the European sea bass, a fish with mixed genetic and environmental sex determination. BMC Genomics. 2015;16:679. 26338702 10.1186/s12864-015-1862-0 PMC4560065

[36] Díaz N, Ribas L, Piferrer F. The relationship between growth and sex differentiation in the European sea bass (Dicentrarchus labrax). Aquaculture. 2013;408–9.

[37] Mazabraud A, Wegnez M, Denis H. Biochemical research on oogenesis. RNA accumulation in the oocytes of teleosts. Dev Biol. 1975;44:326–32. 1132596 10.1016/0012-1606(75)90403-0

[38] Bir J, Rojo-Bartolomé I, Lekube X, Diaz de Cerio O, Ortiz-Zarragoitia M, Cancio I. High production of transfer RNAs identifies the presence of developing oocytes in ovaries and intersex testes of teleost fish. Mar Environ Res. 2023;186 February. 10.1016/j.marenvres.2023.105907 36774708

[39] Desvignes T, Batzel P, Sydes J, Eames BF, Postlethwait JH. MiRNA analysis with prost! Reveals evolutionary conservation of organ-enriched expression and post-transcriptional modifications in three-spined stickleback and zebrafish. Sci Rep. 2019;9:3913. 30850632 10.1038/s41598-019-40361-8 PMC6408482

[40] Love MI, Huber W, Anders S. Moderated Estimation of fold change and dispersion for RNA-seq data with DESeq2. Genome Biol. 2014;15:1–21. 10.1186/s13059-014-0550-8 PMC4302049 25516281

[41] Livak KJ, Schmittgen TD. Analysis of relative gene expression data using Real-Time quantitative PCR and the 2 – ∆∆CT method. Methods. 2001;25:402–8. 11846609 10.1006/meth.2001.1262

[42] Schmittgen TD, Livak KJ. Analyzing real-time PCR data by the comparative CT method. Nat Protoc 2008 36. 2008;3:1101–8. 10.1038/nprot.2008.73 18546601

[43] Desvignes T, Beam MJ, Batzel P, Sydes J, Postlethwait JH. Expanding the annotation of zebrafish MicroRNAs based on small RNA sequencing. Gene. 2014;546:386–9. 24835514 10.1016/j.gene.2014.05.036 PMC4130647

[44] Peichel CL, McCann SR, Ross JA, Naftaly AFS, Urton JR, Cech JN, et al. Assembly of the threespine stickleback y chromosome reveals convergent signatures of sex chromosome evolution. Genome Biol. 2020;21:1–31. 10.1186/s13059-020-02097-x PMC7368989 32684159

[45] Pasquier J, Cabau C, Nguyen T, Jouanno E, Severac D, Braasch I et al. Gene evolution and gene expression after whole genome duplication in fish: the phylofish database. BMC Genomics. 2016;17. 10.1186/s12864-016-2709-z PMC4870732 27189481

[46] Wen M, Pan Q, Jouanno E, Montfort J, Zahm M, Cabau C, et al. An ancient truncated duplication of the anti-Müllerian hormone receptor type 2 gene is a potential conserved master sex determinant in the Pangasiidae catfish family. Mol Ecol Resour. 2022;22:2411–28. 35429227 10.1111/1755-0998.13620 PMC9555307

[47] Gay S, Bugeon J, Bouchareb A, Henry L, Delahaye C, Legeai F, et al. MiR-202 controls female fecundity by regulating Medaka oogenesis. PLOS Genet. 2018;14:e1007593. 30199527 10.1371/journal.pgen.1007593 PMC6147661

[48] Nanda I, Kondo M, Hornung U, Asakawa S, Winkler C, Shimizu A, et al. A duplicated copy of DMRT1 in the sex-determining region of the Y chromosome of the Medaka, Oryzias latipes. Proc Natl Acad Sci. 2002;99:11778–83. 12193652 10.1073/pnas.182314699 PMC129345

[49] Braasch I, Gehrke AR, Smith JJ, Kawasaki K, Manousaki T, Pasquier J, et al. The spotted Gar genome illuminates vertebrate evolution and facilitates human-teleost comparisons. Nat Genet 2016 484. 2016;48:427–37. 10.1038/ng.3526 PMC4817229 26950095

[50] Ferrara AM, Irwin ER. A standardized procedure for internal sex identification in Lepisosteidae. North Am J Fish Manag. 2001;21:956–61.

[51] Lee SJ, Kim J, Choi EK, Jo E, Cho M, Kim J-H, et al. A chromosome-level reference genome of the Antarctic Blackfin icefish chaenocephalus aceratus. Sci Data. 2023;10:657. 37752129 10.1038/s41597-023-02561-w PMC10522714

[52] Rougeot C, Jacobs B, Kestemont P, Melard C. Sex control and sex determinism study in Eurasian perch, Perca fluviatilis, by use of hormonally sex-reversed male breeders. Aquaculture. 2002;211:81–9.

[53] Curzon AY, Shirak A, Ron M, Seroussi E. Master-Key regulators of sex determination in fish and other Vertebrates—A review. Int J Mol Sci. 2023;24:2468. 36768795 10.3390/ijms24032468 PMC9917144

[54] Gan M, Jing Y, Xie Z, Ma J, Chen L, Zhang S, et al. Potential function of testicular MicroRNAs in Heat-Stress-Induced spermatogenesis disorders. Int J Mol Sci. 2023;24:8809. 37240155 10.3390/ijms24108809 PMC10218599

[55] Houdelet C, Blondeau-Bidet E, Estevez-Villar M, Mialhe X, Hermet S, Ruelle F, et al. Circulating MicroRNAs indicative of sex and stress in the European Seabass (Dicentrarchus labrax): toward the identification of new biomarkers. Mar Biotechnol. 2023;25:749–62. 10.1007/s10126-023-10237-0 37581865

[56] Grimson A, Srivastava M, Fahey B, Woodcroft BJ, Chiang HR, King N, et al. Early origins and evolution of MicroRNAs and Piwi-interacting RNAs in animals. Nature. 2008;455:1193–7. 18830242 10.1038/nature07415 PMC3837422

[57] Hertel J, Bartschat S, Wintsche A, Otto C, of the Bioinformatics Computer Lab TS, Stadler PF. Evolution of the let-7 microRNA Family. RNA Biol. 2012;9:231–41. 10.4161/rna.18974 PMC3384580 22617875

[58] Li G, Zhao Y, Wen L, Liu Z, Yan F, Gao C. Identification and characterization of MicroRNAs in the spleen of common carp immune organ. J Cell Biochem. 2014;115:1768–78. 24819892 10.1002/jcb.24843

[59] Gu Y, Zhang L, Chen X. Differential expression analysis of Paralichthys Olivaceus MicroRNAs in adult ovary and testis by deep sequencing. Gen Comp Endocrinol. 2014;204:181–4. 24861804 10.1016/j.ygcen.2014.05.019

[60] Khanehzad M, Nourashrafeddin SM, Abolhassani F, Kazemzadeh S, Madadi S, Shiri E, et al. MicroRNA-30a-5p promotes differentiation in neonatal mouse spermatogonial stem cells (SSCs). Reprod Biol Endocrinol. 2021;19:1–14. 34108007 10.1186/s12958-021-00758-5 PMC8188658

[61] Arefnia M, Motovali-Bashi M, Javadirad SM, Norioun H. Overexpression of hsa-miR-30a-5p and non-obstructive azoospermia: A case-control study. Int J Reprod Biomed. 2022;20:399. 35911855 10.18502/ijrm.v20i5.11054 PMC9334892

[62] Tao W, Sun L, Shi H, Cheng Y, Jiang D, Fu B, et al. Integrated analysis of MiRNA and mRNA expression profiles in tilapia gonads at an early stage of sex differentiation. BMC Genomics. 2016;17:328. 27142172 10.1186/s12864-016-2636-z PMC4855716

[63] Zhao N, Jia L, He X, Zhang B. Sex bias MiRNAs in Cynoglossus semilaevis could play a role in transgenerational inheritance. Comp Biochem Physiol Part D Genomics Proteom. 2021;39:100853. 10.1016/j.cbd.2021.100853 33992844

[64] Matsuda M, Matsuda C, Hamaguchi S, Sakaizumi M. Identification of the sex chromosomes of the Medaka, *Oryzias latipes*, by fluorescence in situ hybridization. Cytogenet Cell Genet. 1998;82:257–62. 9858830 10.1159/000015113

[65] Liew WC, Bartfai R, Lim Z, Sreenivasan R, Siegfried KR, Orban L. Polygenic sex determination system in zebrafish. PLoS ONE. 2012;7:e34397. 22506019 10.1371/journal.pone.0034397 PMC3323597

[66] Jing J, Wu J, Liu W, Xiong S, Ma W, Zhang J et al. Sex-biased MiRNAs in gonad and their potential roles for testis development in yellow catfish. PLoS ONE. 2014;9. 10.1371/journal.pone.0107946 PMC4168133 25229553

[67] Pfennig F, Standke A, Gutzeit HO. The role of Amh signaling in teleost fish – Multiple functions not restricted to the gonads. Gen Comp Endocrinol. 2015;223:87–107. 26428616 10.1016/j.ygcen.2015.09.025

[68] Pisano F, Altomare C, Cervio E, Barile L, Rocchetti M, Ciuffreda MC, et al. Combination of miRNA499 and miRNA133 exerts a synergic effect on cardiac differentiation. Stem Cells. 2015;33:1187–99. 25534971 10.1002/stem.1928 PMC4409033

[69] Chen Z, Xu L, Ye X, Shen S, Li Z, Niu X, et al. Polymorphisms of MicroRNA sequences or binding sites and lung cancer: A Meta-Analysis and systematic review. PLoS ONE. 2013;8:e61008. 23613771 10.1371/journal.pone.0061008 PMC3628762

[70] Ahmed SA, Zhang B, Abdel-Rahman AA. Estrogen-mediated mitigation of cardiac oxidative stress in ovariectomized rats is associated with upregulated cardiac circadian clock Per2 and heart-specific MiRNAs. Life Sci. 2023;331:122038. 37619835 10.1016/j.lfs.2023.122038 PMC10528738

[71] Nachtigall PG, Dias MC, Pinhal D. Evolution and genomic organization of muscle MicroRNAs in fish genomes. BMC Evol Biol. 2014;14:196. 25253178 10.1186/s12862-014-0196-x PMC4177693

[72] Duran BOdaS, Fernandez GJ, Mareco EA, Moraes LN, Salomão RAS, de Gutierrez T, et al. Differential MicroRNA expression in Fast- and Slow-Twitch skeletal muscle of *Piaractus mesopotamicus* during growth. PLoS ONE. 2015;10:e0141967. 26529415 10.1371/journal.pone.0141967 PMC4631509

[73] Nachtigall PG, Dias MC, Carvalho RF, Martins C, Pinhal D. MicroRNA-499 expression distinctively correlates to target genes sox6 and rod1 profiles to resolve the skeletal muscle phenotype in nile tilapia. PLoS ONE. 2015;10:e0119804. 25793727 10.1371/journal.pone.0119804 PMC4368118

[74] Bhuiyan SS, Kinoshita S, Wongwarangkana C, Asaduzzaman M, Asakawa S, Watabe S. Evolution of the myosin heavy chain gene MYH14 and its intronic MicroRNA miR-499: muscle-specific miR-499 expression persists in the absence of the ancestral host gene. BMC Evol Biol. 2013;13:142. 24059862 10.1186/1471-2148-13-142 PMC3716903

[75] Zheng K, Hu F, Zhou Y, Zhang J, Zheng J, Lai C, et al. miR-135a-5p mediates memory and synaptic impairments via the Rock2/Adducin1 signaling pathway in a mouse model of Alzheimer’s disease. Nat Commun. 2021;12:1903. 33771994 10.1038/s41467-021-22196-y PMC7998005

[76] Kadkhoda S, Eslami S, Mahmud Hussen B, Ghafouri-Fard S. A review on the importance of miRNA-135 in human diseases. Front Genet. 2022;13. 10.3389/fgene.2022.973585 PMC9486161 36147505

[77] Herkenhoff ME, Oliveira AC, Nachtigall PG, Costa JM, Campos VF, Hilsdorf AWS, et al. Fishing into the MicroRNA transcriptome. Front Genet. 2018;9:MAR. 10.3389/fgene.2018.00088 PMC5868305 29616080

[78] Xiao J, Zhong H, Zhou Y, Yu F, Gao Y, Luo Y, et al. Identification and characterization of MicroRNAs in ovary and testis of nile tilapia (*Oreochromis niloticus*) by using Solexa sequencing technology. PLoS ONE. 2014;9:e86821. 24466258 10.1371/journal.pone.0086821 PMC3900680

[79] Farlora R, Valenzuela-Miranda D, Alarcón-Matus P, Gallardo-Escárate C. Identification of MicroRNAs associated with sexual maturity in rainbow trout brain and testis through small RNA deep sequencing. Mol Reprod Dev. 2015;82:651–62. 25983107 10.1002/mrd.22499

[80] Zhang D, Shi B, Shao P, Shao C, Wang C, Li J, et al. The identification of MiRNAs that regulate ovarian maturation in *Cynoglossus semilaevis*. Aquaculture. 2022;555:738250.

[81] Guiguen Y, Fostier A, Piferrer F, Chang CF. Ovarian aromatase and estrogens: A pivotal role for gonadal sex differentiation and sex change in fish. Gen Comp Endocrinol. 2010;165:352–66. 19289125 10.1016/j.ygcen.2009.03.002

