# Supplementary material for: Identification of sex-biased MiRNA markers informative of heat-past events
Source: BMC Genomics. 2025 May 8;26:455. doi: 10.1186/s12864-025-11551-8 (PMC12060346; doi:10.1186/s12864-025-11551-8)
Supplement: Supplementary file 6 — Supplementary Material 6: Supplementary Figures [file 12864_2025_11551_MOESM6_ESM.pdf]

Identification of sex-biased miRNA markers informative of heat-past events

Tosca A. van Gelderen[1, 2], Jerome Montfort[3], José Antonio Álvarez-Dios[4], Francesc Piferrer[1], Julien Bobe[3], Laia Ribas[1]

[1] Institut de Ciències del Mar, Consejo Superior de Investigaciones Científicas (ICM-CSIC), 08003, Barcelona, Spain

[2] PhD Program in Genetics, Autonomous University of Barcelona, 08193, Bellaterra, Spain

[3]INRAE, Laboratoire de Physiologie et Génomique des Poissons, Rennes, France

[4] Departamento de Matemática Aplicada, Facultad de Matemáticas, Universidad de Santiago de Compostela, 15781, Santiago de Compostela, Spain.

[*]corresponding author: lribas@icm.csic.es

Supplementary Information

Supplementary Figure 1. Principal Component Analysis (PCA) of miRNA expression of gonadal samples (A, all samples, B, ovaries and C, testes) of European sea bass after heat treatments during early development. Abbreviations: FCT, female control temperature; FHT, female high temperature; MCT, male control temperature; MHT, male high temperature.

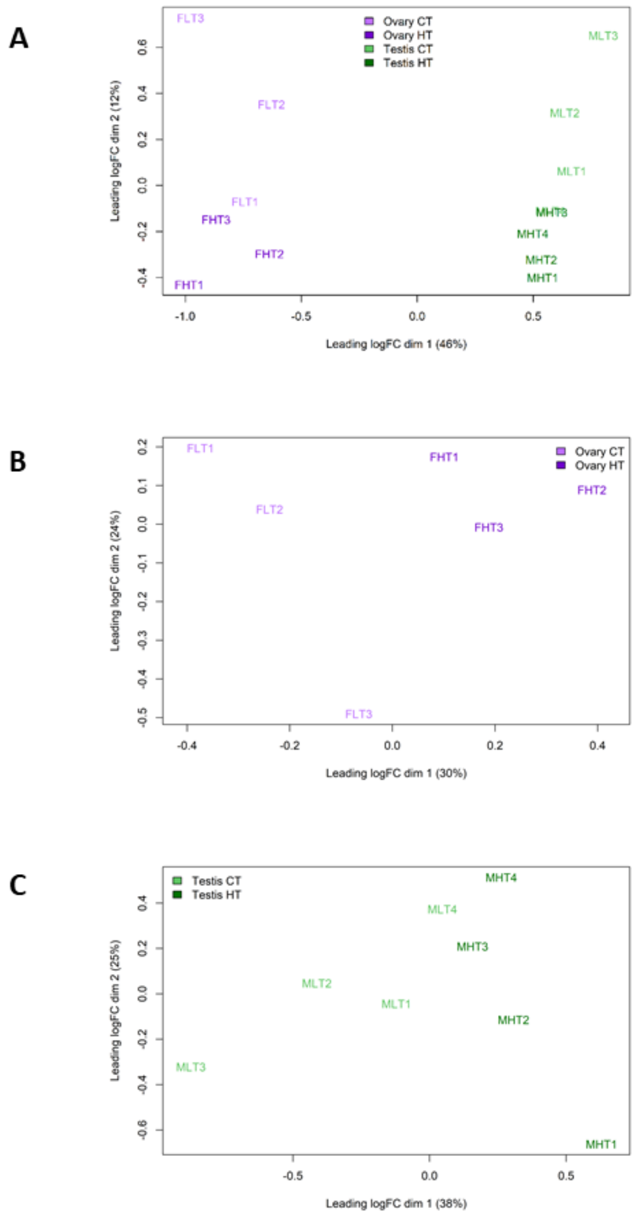

Supplementary Figure 2. Number of targets genes related to Reproduction process Gene Ontology term for each of the miRNA identified in heat-treated compared to control in males. See Dataset 3 for more information.

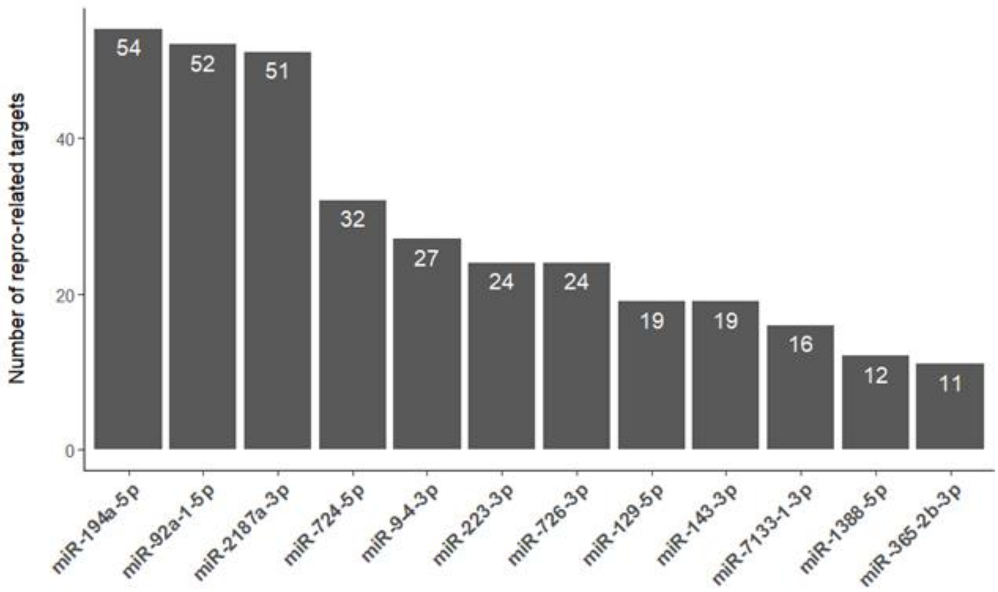

Supplementary Figure 3. Evolutionary relation between the ten teleost fish species used in the present study generated by PhyloT (v2) website tool. https://phylot.biobyte.de/.

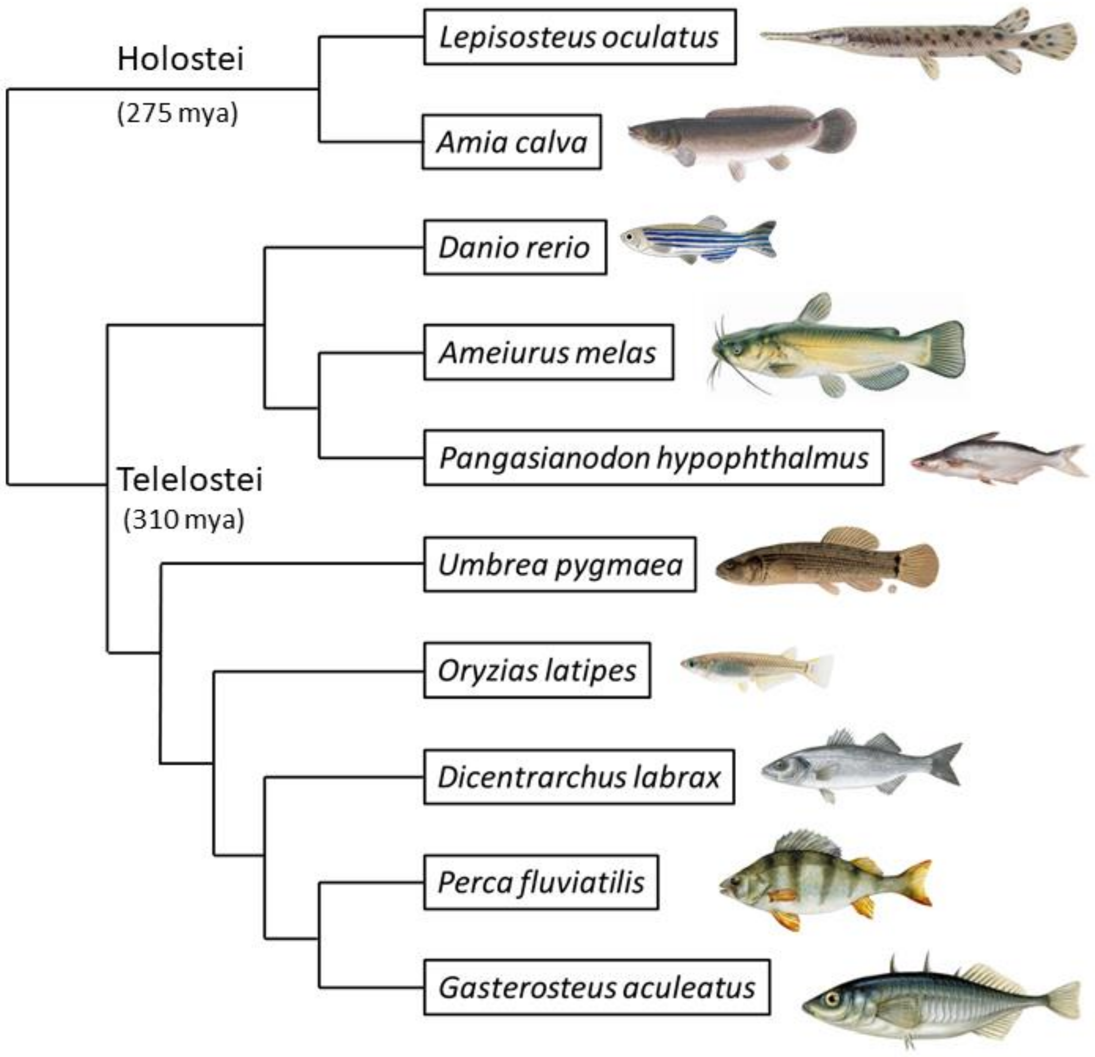

Supplementary Figure 4. Validation of the miRNA sequencing data by quantitative PCR (qPCR) of twelve selected sequenced miRNAs on the ovaries (N=6) and testes (N=8) samples used in the present study. Table shows the results of the two techniques used by comparing the log2FC as obtained by qPCR and RNA-seq for 12 miRNAs. The qPCR data were significant for 10 out of the 12 studied miRNAs. A Student t test was done for qPCR data, while for correlation analysis a Pearson correlation. The comparison of the data was done as follows: ola-miR-146a-3p: ovary high temperature (FHT) *vs*. ovary control temperature (FCT), ola-miR-7132b-3p: FHT *vs*. FCT, ola-miR-143-3p: FHT *vs*. FCT, ola-miR-21-5p: testis high temperature (MHT) *vs*. testis control temperature (MCT), ola-miR-26a-2/3-b-5p: MHT *vs*. MCT, ola-miR-199a-5p: MHT *vs*. MCT, ola-miR-199a-3p: MHT *vs*. MCT, ola-223-3p: MHT *vs*. MCT, ola-192-5p: MHT *vs*. MCT, ola-miR-194a-5p:MTHT *vs*. MCT, ola-miR-726-5p: FCT *vs*. MCT, ola-miR-726-3p: FCT *vs*. MCT.

A

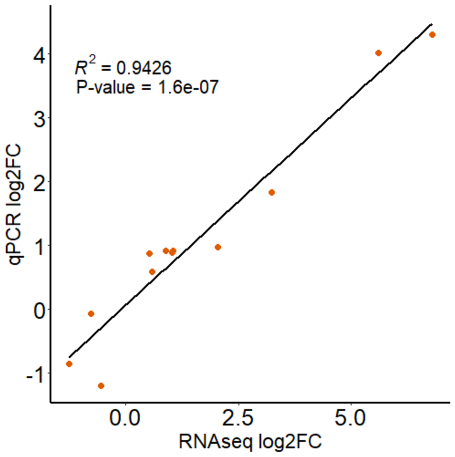

B

miRNA	sample	qPCR	RNAseq
miR-146a-3p	ovary	-0.85	-1.26
miR-7132b-3p	ovary	-0.06	-0.76
miR-143-3p	ovary	-1.19	-0.55
miR-21-5p	testis	0.88	0.53
miR-26a-2/3-b-5p	testis	0.59	0.59
miR-199a-5p	testis	0.92	0.89
miR-199a-3p	testis	0.90	1.03
miR-223-3p	testis	0.91	1.05
miR-192-5p	testis	0.98	2.05
miR-194a-5p	testis	1.83	3.23
miR-726-5p	OvsT	4.02	5.59
miR-726-3p	OvsT	4.31	6.79

Dataset Information

Dataset 1. Prost! output of European sea bass miRNA annotation and reads obtained after miRNA-sequencing of ovaries and testes treated with high temperature during early stages of development.

Dataset 2. Differentially expressed miRNAs in different comparisons of European sea bass gonads treated with high temperature during early gonadal development. Comparisons: male control temperature (MCT) *vs.* female control temperature (FCT), female high temperature (FHT) *vs*. FCT and, MHT *vs.* MCT. The cutoff values were, for adjusted *p*-vales <0.05 and log2FC=0.

Dataset 3. Target genes of male heat-treated differentially expressed miRNAs.

Dataset 4. Dataset to create Figure 3 for the upset plot of conserved seed regions in ovary (A) and testis (B) in ten teleost species analyzed: Danio rerio, Dicentrarchus labrax, Gasterosteus aculeatus, Amia calva, Pangasianodon hypophthalmus, Oryzias latipes, Lepisosteus oculatus, Ameiurus melas, Perca fluviatilis and Umbra pygmaea. In ovary, 210 seed region sequences were conserved in all ten species. In testis, 184 seed region sequences were conserved in all ten species.

Dataset 5. 10 most abundant miRNAs in ovary and testis for each fish species.
